# Item Selection for a New Health-Related Quality of Life Measure for Parkinson's Disease: The Preference-Based Parkinson's Disease Index (PB-PDI)

**DOI:** 10.1155/2023/6559857

**Published:** 2023-01-18

**Authors:** Selina Malouka, Lizabeth Teshler, Nancy Mayo, Marla Beauchamp, Julie Richardson, Ayse Kuspinar

**Affiliations:** ^1^School of Rehabilitation Science, McMaster University, Hamilton, ON L8S 4L8, Canada; ^2^Arts and Science Program, McMaster University, Hamilton, ON L8S 4L8, Canada; ^3^Center for Outcomes Research and Evaluation, McGill University Health Centre- Research Institute, Montreal, QC H3A 0G4, Canada; ^4^School of Physical and Occupational Therapy, McGill University, Montreal, QC H3G 1Y5, Canada; ^5^Department of Health Research Methods, Evidence, and Impact, McMaster University, Hamilton, ON L8S 4L8, Canada

## Abstract

**Background:**

Parkinson's disease (PD) is a neurodegenerative condition, predominantly affecting older adults. Preference-based measures (PBMs) can be used to make decisions about the cost-utility of different treatments. There are currently no PBMs for health-related quality of life (HRQoL) for PD. A previous study identified important health domains for individuals with PD and developed an item pool from existing measures per domain. The current study aims to contribute to the development of a new disease-specific PBM of HRQoL for PD by reducing the current pool of items according to the preferences of individuals with PD.

**Methods:**

Fifty-three participants completed a visual analogue scale (VAS) of self-perceived health, the prototype PBM measure, and an item importance rating. To reduce the item pool, the following were calculated: (1) inter-item correlations; (2) impact of each item based on item performance and importance rating; (3) directionality of response options by comparing the VAS scores against each item.

**Results:**

Participants (male = 54.7%, age = 60.0 ± 10.2) had a median Hoehn and Yahr score of 2.5 (interquartile range = 1). Items supported for inclusion by this analysis were sleep, fatigue, tremor, mood, walking, memory, and dexterity. Items demonstrating a logical decrease in VAS score with each increasing severity level were sleep, memory, tremor, fatigue, and mood.

**Conclusion:**

This PBM will be critical for informing decisions about the cost-utility of PD treatments, guiding the resource allocation within our healthcare system. Future research will include cognitive debriefing with individuals with PD to refine item response options.

## 1. Background

Parkinson's disease (PD) is a progressive neurological condition that primarily impacts motor function [1]. It is estimated that 9.4 million people have PD worldwide [2] and 100,000 people in Canada [3]. As there is no cure for PD, individuals must undergo life-long treatment including pharmacological, therapeutic, or surgical interventions [4], placing a financial burden on the healthcare system and the individual [4, 5]. As such, the need for standardized tools to assess the cost-effectiveness of different interventions is paramount [6].

A common method for the economic evaluation of interventions is cost-utility analysis, where the incremental cost of an intervention is compared to its incremental health improvement expressed in quality adjusted life years (QALYs). Closely tied to QALYs are the quality of life (QoL) and health-related quality of life (HRQoL). Quality of life refers to an “individual” perception of their position in life in the context of the culture in which they live and in relation to their goals, expectations, standards, and concerns” [7], while HRQoL refers to the “aspects of self-perceived well-being that are related to or affected by the presence of disease or treatment” [8].

Preference-based measures (PBMs) are used to calculate QALYs and assess the cost-utility of interventions [9]. They attach a value to each domain of health [10] and have the advantage of producing a single number (anchored from 0 to 1) as the final score, balancing gains in one domain against losses in another [10]. The EuroQOL 5-dimension 5-level (EQ-5D-5L) and Health Utilities Index Mark 3 (HUIIII) are PBMs that can be used for cost-utility analyses, however; these are generic measures and may not optimally reflect the health concerns of people with PD [6]. Specifically, the importance assigned to the various QoL areas is based on the public's opinions and not on those with PD, who may have very different priorities. Together, this may make generic PBMs unresponsive to change among individuals with PD [6, 11]. Currently, no PBM of HRQoL exists for PD [11].

Previous studies have identified the health domains that are important to individuals with PD, as well as twelve prototype items that are most reflective of these domains (i.e., sleep, fatigue, tremor, mood, walking, memory, dexterity, urine control, concentration, speech, freezing, and swallowing) [12, 13]. The twelve prototype items were then refined in an item writing workshop consisting of a panel of experts [14]. The next step in the development of a PBM is to select the most relevant items for inclusion in the measure. Preference-based measures are unique in that they rarely include more than eight items, with one item per dimension [15]. This is because one item per dimension can generate many health states, calculated as the number of response options to the power of the number of items. Furthermore, the number of health states is limited by how many states participants can address during preference elicitation tasks [15]. As such, the purpose of this study is to assess and select items for inclusion in a PBM for PD, contributing to the development of a new multidimensional disease-specific PBM of HRQoL for people with PD: the Preference-Based Parkinson's Disease Index (PB-PDI).

## 2. Methods

### 2.1. Study Design

This was a cross-sectional study with primary data collection.

### 2.2. Sample

Participants were recruited from the Quebec Parkinson Network (QPN) registry (*n* = 1,041). The research team contacted potential participants by telephone. If individuals were interested, a link to an online survey was provided via E-mail, which included the consent form and the study survey. The online survey is available in both English and French. The sampling strategy was purposeful to ensure that the participants contacted were diverse in terms of age, disease severity, and sex. Bilingual individuals (*n* = 472) were extracted from the registry and split by sex (males *n* = 330, females *n* = 142). Within each sex category, individuals were split into age groups using 10-year increments. Within each age group, persons with varying disease severity were contacted. Out of the seventy-four individuals who consented to be e-mailed the survey link, fifty-three individuals completed it (i.e., a 71.6% response rate).

### 2.3. Outcome Measures

The following outcome measures were administered through an online survey format using LimeSurvey software, and were available in English and French.

#### 2.3.1. Demographic Questionnaire

The demographic questionnaire asked about participants' sex, age, language fluency, geographical location, education, marital status, living situation, employment status, household income, year of diagnosis, presence of chronic conditions, and if the survey was completed independently or with help.

#### 2.3.2. Self-Assessment Parkinson's Disease Disability Scale (SPDDS)

The SPDDS is a disease-specific questionnaire consisting of 24 items asking about the individual performance of activities in daily living [16]. Participants are asked to rate the degree of difficulty they have performing each of the identified activities in general, without the use of a mobility aid. Each item can be answered on a 5-point Likert scale from 1 = “able to do alone and without difficulty” to 5 = “unable to do at all.” The total score ranges from 24 to 120, with higher scores suggesting a higher severity of impairment.

#### 2.3.3. Preference-Based Parkinson's Disease Index (PB-PDI) and Importance Rating

The development of the PB-PDI is described in the following section. There was a total of twelve items in the PB-PDI, which asked about participants' sleep, tremors, memory, urine control, mood, fatigue, swallowing, walking, concentration, speech, dexterity, and freezing due to PD during the last two-week period (Supplementary Table 1). Participants answered on an ordinal response option scale ranging from 1 to 3, with 3 being the worst health state. Following each PB-PDI item, participants were asked to rate how important each item was to their QoL on a 5-point Likert scale from 1 = unimportant to 5 = extremely important.

#### 2.3.4. Visual Analogue Scale (VAS) of Self-Perceived Health

Participants were asked to evaluate their general health state by using a visual analogue scale (VAS) from 0 = “worst imaginable health state” to 100 = “best imaginable health state.”

#### 2.3.5. The Hoehn and Yahr Scale (H&Y)

This scale is used to categorize the severity of PD from I (mild symptoms on one side of the body) to V (full helplessness). It has a moderate to significant inter-rater reliability, with nonweighted and weighted kappa scores ranging between 0.44 and 0.71 [17]. Further psychometric testing of the H&Y scale is limited [17]. Participants' H&Y stages were extracted from the QPN registry and only used during the purposeful sampling.

### 2.4. Development of the PB-PDI

#### 2.4.1. Item Generation

Item generation was not within the scope of this paper; the prototype items were already been developed before the start of this project. In previous work, people with PD were asked to identify the most important domains of their lives that were affected by PD [12]. Next, items relevant to each domain were collected from existing measures to form an item pool. One item per domain was selected using Rasch analysis [13], and an item writing workshop was conducted to further refine and translate items into French [14].

#### 2.4.2. Item Selection

Item selection for the PB-PDI was the purpose of the current study. Items were selected for inclusion in the PB-PDI based on the following statistical and clinical criteria:

1. A high cross-product of importance and prevalence (i.e., impact score)
2. Inter-item correlations below 0.7 to reduce redundancy [18] and
3. Evidence from the literature that the health domain is responsive to treatment or is a side-effect of it

#### 2.4.3. Ordering of Response Options

Participants rated their level of functioning on the ordinal response options of the PB-PDI, and scored on a range of 1 to 3, with 3 being the worst health state. To assess the logical ordering of the response options with respect to the health rating, the score of each item was compared to the VAS. The logical ordering of these response options would be indicated by the VAS scores showing decreasing values as response option severity increases from 1 (least severe) to 3 (most severe).

### 2.5. Statistical Analysis

Descriptive summary statistics were presented as means and standard deviations (SD) for continuous variables. Medians and interquartile ranges (IQR), or percentages where applicable were presented for categorical data. The distribution of the sample over each response level (i.e., levels 1 –3) on the PB-PDI and the distribution of responses over importance rating levels (i.e., not important to extremely important) were calculated as percentages.

The mean impact score of each item on the PB-PDI was calculated as the product of the prevalence of participants who were affected by each health domain (i.e., those scoring a level 2 or higher on the PB-PDI) and the mean importance rating for that item [19]: impact = (% scoring a level 2+) *∗* (mean importance rating for level 2+)/100. Similar exploratory analyses were conducted by disease duration (i.e., >5 years and ≤5 years since diagnosis) [20] to confirm the selection of items. Next, inter-item correlations were calculated using polychoric correlations. Lastly, the directionality of response options was evaluated by calculating, for each response option, the mean (SD) values on the VAS. All statistical tests were conducted using STATA 16.0 (STATACorp LLC, College Station, Texas).

### 2.6. Sample Size

To conduct correlation analyses (*α* = 0.05, *β* = 0.2, and an expected *r* = 0.7 between the items [i.e., the correlation cut-off to reduce redundancy]), the required sample size would be *n* = 13. To conduct impact score analyses, the parameter of interest is the proportion of people with PD who rate items as very important or higher. With 50 participants, the 95% confidence interval around proportions of 50% would be ±13%. As such, we aimed to recruit a minimum of 50 participants.

## 3. Results

Table 1 outlines the characteristics of the entire sample (*n* = 53). Participants were predominantly male (54.7%), and the mean age of the sample was 60.0 years (SD = 10.2), ranging from 36 to 79 years. The majority of the sample had a postsecondary education (92.5%) and a chronic condition in addition to PD (62.3%). The sample had a mean SPDDS score of 36.7 (SD = 11.8), a mean VAS score of 66.6 (SD = 21.6), a median H&Y score of 2.5 (IQR = 1), and a mean of 8.3 years (SD = 6.1) since their PD diagnosis.

Participants answered at all item response levels (Supplementary Table 1). In terms of the distribution of responses over the importance rating levels, most participants rated all items as very important (response level 4) or extremely important (response level 5) to their overall QoL (Supplementary Table 2). Eighty-seven percent of the sample identified walking as very or extremely important, followed by sleep (83.0%), memory (79.2%), and fatigue (79.2%).

Table 2 presents the mean impact score for the whole sample. Items with the highest prevalence were sleep (81.1%), tremor (79.2%), fatigue (77.4%), dexterity (64.2%), mood (62.3%), and memory (60.4%). Moreover, the impact score was calculated for each item and used to rank them from the highest to the lowest. The top six items with the highest impact score were sleep (3.5), fatigue (3.2), tremor (3.2), mood (2.6), walking (2.4), and memory (2.4).

Table 3 reports the exploratory subgroup analysis by disease duration. The exploratory analysis supported the same top six items of sleep, fatigue, tremor, mood, walking, and memory for males and for those with >5 years since their PD diagnosis. For those with ≤5 years since their PD diagnosis, dexterity was ranked slightly higher than walking. Items that consistently demonstrated the lowest impact score for the whole sample and by most subgroups were swallowing, freezing, speech, and concentration. These items qualified for exclusion from the PB-PDI.

The polychoric correlation matrix for each item on the PB-PDI showed that concentration correlated highly with both memory and fatigue (*r* = 0.7), indicating redundancy between these items (Supplementary Table 3). As a result, the item on concentration qualified for exclusion from the PB-PDI because it was highly correlated with two other items and had the lower impact score.

For the last item inclusion criterion, systematic reviews specific to the PD population were consulted when available. The criterion was fulfilled by all the items, as the literature suggested that all selected health domains were responsive to treatment, whether it is therapeutic, pharmacological, or surgical [21–30].

Table 4 presents the mean VAS rating for each item's response options. The items that demonstrated a logical decrease in VAS score with each increasing severity level were sleep, memory, tremors, freezing, fatigue, concentration, and mood. Items that did not demonstrate a logical decrease in VAS score with each increasing severity level were walking, dexterity, urine control, swallowing, and speech.

## 4. Discussion

The overall purpose of this study was to select and assess items for inclusion in a new multidimensional disease-specific PBM of HRQoL for people with PD: the PB-PDI. Previously, our research group identified the health domains that are important to individuals with PD [12], determined prototype items to capture these domains [13], and refined the prototype items based on the opinions of an expert panel [14]. The result was twelve items proposed for consideration in the new PBM. The current study identified seven items for inclusion in the final PB-PDI, which were sleep, fatigue, tremor, mood, walking, memory, and dexterity.

The first criterion for item selection was a high ranking of items based on impact scores, as calculated by the product of item prevalence and importance rating. The impact method was chosen as it is an established way of item reduction [19]. Relative to psychometric methods such as factor analysis, the impact method ensures that health domains that are both important and prevalent among patients are represented in items which could have otherwise been excluded [19]. A majority of participants rated all items as very important or extremely important, and the prevalence of each item ranged from 22.6% to 81.1%. This resulted in the highest impact scores for sleep, fatigue, tremor, mood, walking, and memory. Items that demonstrated the lowest impact scores, both for the whole sample and by subgroup, were swallowing, freezing, speech, and concentration.

The second criterion for item selection was removing items with inter-item correlations above 0.7. This criterion, commonly used together with the impact method [31], was chosen to ensure that there is no redundancy between the items, assisting with further item reduction. Concentration correlated highly with memory and fatigue; therefore, it was excluded given that it had a lower impact score.

Lastly, items had to have evidence that the health domain was responsive to treatment or was a side-effect of it. All domains were evidenced to be responsive to pharmacological, therapeutic, or surgical treatment or a combination [21–30]. For example, sleep issues can be managed pharmacologically using Modafinil [22] or therapeutically using exercise [23]. Similarly, walking can be managed pharmacologically using dopamine agonists [25] and therapeutically with exercise [24]. Health domains were included if they were also potential side effects of treatments, such as fatigue [32].

In descriptively assessing the directionality of response options for the seven selected items, the items demonstrating a logical decrease in VAS score with each increasing severity level were sleep, memory, tremor, fatigue, and mood. Of those items, tremor and mood demonstrated near equal spacing between the response options (i.e., potential linearity). Items that have been chosen for inclusion in the PB-PDI and did not demonstrate appropriate directionality of response options will undergo cognitive debriefing with individuals with PD. These items were walking and dexterity.

The current study had several strengths and limitations. First, using the QPN participant registry provided access to a comprehensive list of participants. However, participants' information in this registry, such as the H and Y scores, could have been outdated. As such, disease duration was used instead of H&Y scores for the subgroup analysis as this data was recently collected. Furthermore, information regarding the racial makeup of the sample was not collected. Second, the current study methodology employed an online survey. A strength of this survey is that it was offered in both English and French to accommodate for a larger demographic. However, the online format could have discouraged participation and limited responses to participants from a specific socioeconomic status. Lastly, since participants rated all items very highly, more weight was given to the prevalence of health issues in the calculation of the impact scores. Although the sample was representative with regards to age and sex, it may not have been representative of the full disease spectrum of PD; the sample was highly functioning as evidenced by the health descriptors in Table 1. As such, impact scores are representative of a primarily high- functioning group of individuals with PD. This could have led to the exclusion of items that are more relevant at later stages of the disease (e.g., swallowing), limiting the usage of the PB-PDI for HRQoL assessments in community-dwelling, higher-functioning individuals with PD. Lastly, the subgroup analysis was not sufficiently powered; however, it allowed for the exploration of items that could have been missed in our overall analysis.

Overall, this study has important implications for addressing the critical need for a disease-specific HRQoL PBM for PD [11]. Generic PBMs of HRQoL have been criticized for not optimally reflecting the health concerns of people with PD [6]. For example, commonly used generic PBMs such as the EQ-5D-5L, HUI II, HUI III, and Short Form 6-Dimensions (SF-6D) are missing items that were nominated as important by people with PD [12]. Consequently, these generic measures may not be as responsive to changes in the PD population [6]. However, the proposed PB-PDI is advantageous as it includes PD-specific items that are absent from generic PBMs, such as sleep, fatigue, tremor, and dexterity.

Preference-based measures are becoming increasingly popular given their advantages in assessing the cost-utility of interventions through the calculation of QALYs [9]. QALYs are advantageous in that they combine the quality and quantity of life into a single number which can be compared across interventions. Given that PD involves a variety of life-long management strategies [4], it is important for researchers and policymakers to make decisions about how to allocate resources accordingly. As generic PBMs do not capture the health domains that are most important to individuals with PD, it is essential to develop a disease-specific PBM to be used in the cost-utility assessments of PD interventions. The current study contributed to the development of such a measure, and the proposed PB-PDI can be used by researchers and policymakers to make decisions about appropriate treatment plans, monitor their effectiveness [9], and evaluate new and emerging treatment options in the PD population.

In conclusion, the results of this study supported the inclusion of seven items in the PB-PDI. The next steps in the development of the PB-PDI include (1) cognitive debriefing with individuals with PD to further refine the selected items; (2) elicitation of preference weights and development of a scoring algorithm; (3) evaluation of the PB-PDI's construct validity.

## Figures and Tables

**Table 1:** Characteristics of the sample (*n* = 53).

Characteristic	Mean (SD) or *N* (%)
Participant characteristics	
Sex	
Male	29 (54.7)
Age (years)	60.0 (10.2)
Language fluency	
English	23 (43.4)
French	28 (52.8)
Other	2 (3.8)
Geographical location	
Quebec	53 (100)
Education level	
Less than high school	1 (1.9)
High school	3 (5.7)
CEGEP or college	14 (26.4)
Bachelor's degree	14 (26.4)
Graduate degree	21 (39.6)
Household income	
Less than $20,000	3 (5.9)
≥$20,000 but <$50,000	7 (13.7)
≥$50,000 but < $100,000	19 (37.3)
≥$100,000 but < $150,000	15 (29.4)
≥ $150,000	7 (13.7)
Marital status	
Married/common law	38 (71.7)
Divorced/separated	10 (18.9)
Widowed	0
Never married	5 (9.4)
Living situation	
Your own home	47 (90.4)
Assisted living	0
Nursing home or long-term care	0
Retirement facility	1 (1.9)
Other	4 (7.7)
Employment status	
Full-time	7 (13.5)
Part-time	2 (3.9)
Self-employed	1 (1.9)
Student	0
Short-term disability	3 (5.8)
Long-term disability	9 (17.3)
Retired	29 (55.8)
Unemployed	0
Other	1 (1.9)
Participation in therapeutic trial	
Yes	3 (5.8)
No	49 (94.2)

Participant health descriptors	
Presence of chronic disease	
Yes	33 (62.3)
No	20 (37.7)
SPDDS score	36.7 (11.8)
H&Y score^a^	2.5 (1)
VAS score for self-perceived health	66.6 (21.6)
Years since PD diagnosis	8.3 (6.1)

Survey characteristics	
Language of completion	
English	30 (56.6)
French	23 (43.4)
Format of completion	
On their own	49 (92.5)
Assistance of an informal caregiver	4 (7.6)
Assistance of a formal caregiver	0

^a^Median score and interquartile ranges (IQR) reported. SD, standard deviation; N, number; SPDDS, self-assessment Parkinson's disease disability scale; H&Y, Hoehn and Yahr; VAS, visual analogue scale; PD, Parkinson's disease.

**Table 2:** Impact score of each item on the PB-PDI (*n* = 53) for those scoring a level 2 or higher.

Item	% scoring a level 2+	Mean (SD) importance rating for level 2+	Mean impact score
Sleep	81.1	4.3 (0.8)	3.5
Fatigue	77.4	4.1 (0.8)	3.2
Tremor	79.2	4.0 (1.1)	3.2
Mood	62.3	4.1 (0.8)	2.6
Walking	56.6	4.3 (0.6)	2.4
Memory	60.4	4.0 (0.9)	2.4
Dexterity	64.2	3.5 (1.1)	2.2
Urine control	52.8	3.9 (0.9)	2.1
Concentration	49.1	3.9 (0.9)	1.9
Speech	35.8	4.3 (0.7)	1.5
Freezing	34.0	4.4 (0.9)	1.5
Swallowing	22.6	4.0 (1.0)	0.9

Impact = (% scoring a level 2+) *∗* (mean importance rating for level 2+)/100. SD, standard deviation.

**Table 3:** Items ranked from highest to lowest mean impact score for the whole sample, as well as by sex and years since PD diagnosis.

>5 years (*n* = 32)		≤5 years (*n* = 21)	
Item	Mean impact score	Item	Mean impact score
Sleep	3.6	Sleep	3.3
Fatigue	3.3	Tremor	3.1
Tremor	3.2	Fatigue	3.1
Walking	2.7	Mood	2.5
Mood	2.6	Memory	2.2
Memory	2.5	Dexterity	2.2
Concentration	2.5	Walking	2.1
Dexterity	2.3	Urine control	1.8
Urine control	2.3	Speech	1.4
Freezing	1.8	Swallowing	1.3
Speech	1.4	Concentration	1.1
Swallowing	0.6	Freezing	1.1

**Table 4:** Mean (SD) VAS rating for each item response option.

Items	Level	*N* (%)	Mean (SD) VAS rating
Sleep	1	10 (18.9)	71.1 (21.5)
	2	20 (37.7)	69.9 (17.4)
	3	23 (43.4)	61.9 (24.7)

Fatigue	1	12 (22.6)	78.3 (15.7)
	2	38 (71.7)	66.5 (19.2)
	3	3 (5.7)	21.7 (10.4)

Tremor	1	11 (20.8)	77.2 (13.5)
	2	25 (47.2)	67.8 (22.0)
	3	17 (32.1)	58.1 (23.0)

Mood	1	20 (37.7)	81.1 (9.1)
	2	28 (52.8)	61.8 (20.2)
	3	5 (9.4)	36.2 (23.8)

Walking	1	23 (43.4)	71.9 (20.3)
	2	28 (52.8)	62.3 (22.4)
	3	2 (3.8)	66.0 (22.6)

Memory	1	21 (39.6)	73.0 (18.1)
	2	25 (47.2)	62.9 (23.2)
	3	7 (13.2)	61.0 (23.7)

Dexterity	1	19 (35.9)	70.3 (19.2)
	2	33 (62.3)	64.1 (23.0)
	3	1 (1.9)	82.0

Urine control	1	25 (47.2)	65.7 (23.5)
	2	22 (41.5)	68.0 (21.0)
	3	6 (11.3)	65.3 (18.7)

Concentration	1	27 (50.9)	74.8 (16.0)
	2	24 (45.3)	60.5 (22.4)
	3	2 (3.8)	30.0 (28.3)

Speech	1	34 (65.4)	69.7 (20.0)
	2	16 (30.8)	60.5 (25.5)
	3	2 (3.9)	64.5 (20.5)

Freezing	1	35 (66.0)	71.6 (14.8)
	2	17 (32.1)	58.2 (29.4)
	3	1 (1.9)	35.0

Swallowing	1	41 (77.4)	71.1 (20.2)
	2	9 (17.0)	48.0 (21.1)
	3	3 (5.7)	62.0 (15.1)

SD, standard deviation; *N* = number; VAS, visual analogue scale.

## Data Availability

All relevant data are presented in the manuscript and supplementary materials.

## Additional Points

- Preference-based measures (PBMs) can assess different treatments' cost-utility; however, currently no PBM exists for Parkinson's disease (PD).
- We assessed items for inclusion in a PBM using impact and correlation analyses, and items on sleep, fatigue, tremor, mood, walking, memory, and dexterity were included.
- This PBM includes items specific to PD that are missing from generic measures.

## Supplementary Materials

Supplementary Materials One supplementary file with tables presenting (1) the distribution of responses over each response level on the PB-PDI; (2) importance rating of each item on the PB-PDI; and (3) polychoric correlation matrix for each item on the PB-PDI.
