# Supplementary material for: Item Selection for a New Health-Related Quality of Life Measure for Parkinson's Disease: The Preference-Based Parkinson's Disease Index (PB-PDI)
Source: Neurol Res Int. 2023 Jan 18;2023:6559857. doi: 10.1155/2023/6559857 (PMC9876679; doi:10.1155/2023/6559857)
Supplement: Supplementary Materials — One supplementary file with tables presenting (1) the distribution of responses over each response level on the PB-PDI; (2) importance rating of each item on the PB-PDI; and (3) polychoric correlation matrix for each item on the PB-PDI. [file 6559857.f1.docx]

Item Selection for a New Health-Related Quality of Life Measure for Parkinson’s Disease: The Preference-Based Parkinson’s Disease Index (PB-PDI)

Selina Malouka, BKin, ^a^ Lizabeth Teshler, B.Arts Sc., ^b^ Nancy Mayo, PhD, ^c, d^ Marla Beauchamp, PT, PhD, ^a^ Julie Richardson, PT, PhD, ^a, e^ Ayse Kuspinar, PT, PhD ^a^

^a^ School of Rehabilitation Science, McMaster University, Hamilton, ON, L8S 4L8,

Canada.

^b^ Arts and Science program, McMaster University, Hamilton, ON, L8S 4L8, Canada

^c^ Center for Outcomes Research and Evaluation, McGill University Health Centre- Research Institute, Montreal, QC, H3A 0G4, Canada

^d^ School of Physical and Occupational Therapy, McGill University, Montreal, QC, H3G 1Y5, Canada

^e^ Department of Health Research Methods, Evidence, and Impact, McMaster University,

Hamilton, Ontario, L8S 4L8, Canada.

Corresponding Author: Ayse Kuspinar, PT, PhD

School of Rehabilitation Science

McMaster University

1400 Main St. W., IAHS

L8S 1C7

Hamilton, Ontario

Canada

[kuspinaa@mcmaster.ca](mailto:kuspinaa@mcmaster.ca)

**SUPPLEMENTARY MATERIAL**

**Table 1:** Distribution of responses over each response level (i.e., levels 1-3) on the PB-PDI

| Item | Frequency (%) | | |
| --- | --- | --- | --- |
|  | 1 | 2 | 3 |
| Sleep | 10 (18.9) | 20 (37.7) | 23 (43.4) |
| Tremor | 11 (20.8) | 25 (47.2) | 17 (32.1) |
| Memory | 21 (39.6) | 25 (47.2) | 7 (13.2) |
| Urine Control | 25 (47.2) | 22 (41.5) | 6 (11.3) |
| Mood | 20 (37.7) | 28 (52.8) | 5 (9.4) |
| Fatigue | 12 (22.6) | 38 (71.7) | 3 (5.7) |
| Swallowing | 41 (77.4) | 9 (17.0) | 3 (5.7) |
| Walking | 23 (43.4) | 28 (52.8) | 2 (3.8) |
| Concentration | 27 (50.9) | 24 (45.3) | 2 (3.8) |
| Speech | 34 (65.4) | 16 (30.8) | 2 (3.9) |
| Dexterity | 19 (35.9) | 33 (62.3) | 1 (1.9) |
| Freezing | 35 (66.0) | 17 (32.1) | 1 (1.9) |

**Table 2:** Importance rating of each item on the PB-PDI (*n* = 53)

| Item | Frequency (%) | | | | |
| --- | --- | --- | --- | --- | --- |
|  | Not important | Slightly important | Moderately important | Very important | Extremely important |
| Walking | 1 (1.9) | 1 (1.9) | 5 (9.4) | 29 (54.7) | 17 (32.1) |
| Sleep | 0 | 1 (1.9) | 8 (15.1) | 22 (41.5) | 22 (41.5) |
| Memory | 0 | 3 (5.7) | 8 (15.1) | 24 (45.3) | 18 (34.0) |
| Fatigue | 0 | 3 (5.7) | 8 (15.1) | 28 (52.8) | 14 (26.4) |
| Speech | 3 (5.8) | 1 (1.9) | 9 (17.3) | 19 (36.5) | 20 (38.5) |
| Mood | 2 (3.8) | 4 (7.6) | 8 (15.1) | 20 (37.7) | 19 (35.9) |
| Urine Control | 1 (1.9) | 5 (9.4) | 8 (15.1) | 24 (45.3) | 15 (28.3) |
| Concentration | 1 (1.9) | 3 (5.7) | 11 (20.8) | 19 (35.9) | 19 (35.9) |
| Swallowing | 6 (11.3) | 2 (3.8) | 9 (17.0) | 20 (37.7) | 16 (30.2) |
| Freezing | 7 (13.2) | 5 (9.4) | 7 (13.2) | 13 (24.5) | 21 (39.6) |
| Tremor | 3 (5.7) | 2 (3.8) | 14 (26.4) | 15 (28.3) | 19 (35.9) |
| Dexterity | 1 (1.9) | 6 (11.3) | 13 (24.5) | 21 (39.6) | 12 (22.6) |

**Table 3:** Polychoric correlation matrix for each item on the PB-PDI

| Item | 1 | 2 | 3 | 4 | 5 | 6 | 7 | 8 | 9 | 10 | 11 | 12 |
| --- | --- | --- | --- | --- | --- | --- | --- | --- | --- | --- | --- | --- |
| 1 | 1.0 |  |  |  |  |  |  |  |  |  |  |  |
| 2 | 0.2 | 1.0 |  |  |  |  |  |  |  |  |  |  |
| 3 | 0.1 | 0.1 | 1.0 |  |  |  |  |  |  |  |  |  |
| 4 | 0.1 | 0.4 | 0.1 | 1.0 |  |  |  |  |  |  |  |  |
| 5 | 0.1 | -0.1 | -0.2 | 0.2 | 1.0 |  |  |  |  |  |  |  |
| 6 | -0.1 | 0.1 | 0.3 | 0.1 | 0.0 | 1.0 |  |  |  |  |  |  |
| 7 | 0.0 | 0.2 | -0.0 | 0.2 | 0.1 | 0.1 | 1.0 |  |  |  |  |  |
| 8 | 0.1 | 0.2 | 0.1 | -0.1 | 0.3 | 0.1 | 0.5 | 1.0 |  |  |  |  |
| 9 | 0.5 | -0.1 | -0.3 | 0.1 | 0.4 | -0.2 | 0.4 | 0.2 | 1.0 |  |  |  |
| 10 | 0.3 | 0.3 | 0.4 | 0.4 | 0.5 | -0.1 | -0.0 | -0.0 | 0.0 | 1.0 |  |  |
| 11 | 0.3 | 0.4 | **0.7** | 0.4 | 0.1 | -0.1 | 0.2 | 0.3 | 0.3 | **0.7** | 1.0 |  |
| 12 | 0.3 | 0.3 | 0.4 | 0.4 | 0.1 | -0.1 | 0.4 | 0.3 | 0.2 | 0.6 | 0.6 | 1.0 |

Bolded = met the cut-off correlation value of 0.7.

Item 1, dexterity; 2, sleep; 3, memory; 4, tremors; 5, freezing; 6, urine control; 7, swallowing; 8, speech; 9, walking; 10, fatigue; 11, concentration; 12, mood
